# Editorial: Optimizing outcomes for children with immune-mediated chronic kidney disease

**DOI:** 10.3389/fped.2024.1522963

**Published:** 2024-12-03

**Authors:** Thomas Renson, Evelien Snauwaert, Lorraine Hamiwka, Susa Benseler, Lovro Lamot

**Affiliations:** ^1^ Pediatric Rheumatology, Department of Internal Medicine and Pediatrics, Ghent University Hospital, Ghent, Belgium; ^2^ European Reference Network for Rare Immunodeficiency, Autoinflammatory, Autoimmune and Pediatric Rheumatic Diseases, Ghent University Hospital, Ghent, Belgium; ^3^ Pediatric Nephrology, Department of Internal Medicine and Pediatrics, Ghent University Hospital, Ghent, Belgium; ^4^ European Rare Kidney Disease Network, Ghent University Hospital, Ghent, Belgium; ^5^ Nephrology, Department of Pediatrics, University of Calgary, Calgary, AB, Canada; ^6^ Cumming School of Medicine, Alberta Children’s Hospital Research Institute, University of Calgary, Calgary, AB, Canada; ^7^ Children’s Health Ireland, Dublin, Ireland; ^8^ Department of Pediatrics, University of Zagreb School of Medicine, Zagreb, Croatia; ^9^ Nephrology, Dialysis and Transplantation, Department of Pediatrics, University Hospital Center Zagreb, Zagreb, Croatia

**Keywords:** immune-mediated, kidney disease, glomerular disease, CKD—chronic kidney disease, pediatrics

**Editorial on the Research Topic**
Optimizing outcomes for children with immune-mediated chronic kidney disease

Kidney involvement is an important determinant of morbidity and mortality in systemic immune-mediated diseases. Acute kidney injury may transition into chronic kidney disease (CKD) ultimately resulting in kidney failure warranting kidney replacement therapy. The glomerulonephritides (GN) are a heterogeneous group of immune-mediated diseases characterized by glomerular inflammation and injury (1, 2). GN is the most frequent cause of kidney failure in young people, representing a major burden regarding long-term kidney outcomes (3). The different mechanisms of immune dysregulation affecting the kidney are complex and heterogeneous (Figure 1) (4). Auto-antibodies directed against renal antigens can cause direct kidney disease; whereas indirect kidney disease can be induced by systemic autoimmunity, e.g., immune complex formation, alternative pathway complement activation, and the formation of neutrophil extracellular traps. Although there are many pathways of immune-mediated kidney inflammation, pathways leading to CKD are more homogenous (4). A loss of immune homeostasis in the kidney leads to further recruitment of immune cells and damage accrual. An uncoordinated tissue repair subsequently leads to tissue fibrosis. Immune function is severely compromised in kidney failure, leading to a vicious cycle facilitating further kidney disease and damage.

**Figure 1:**
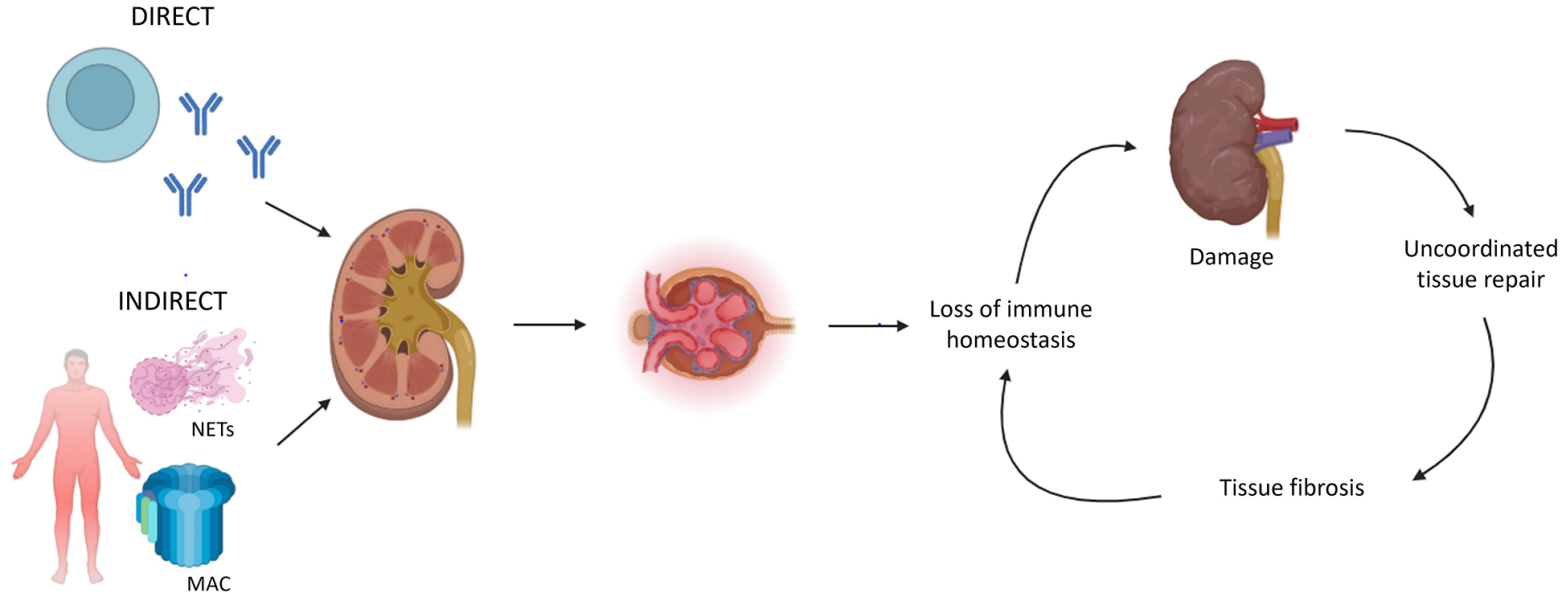
Key mechanisms of immune dysregulation leading to chronic kidney disease (CKD) (4). Immune-mediated kidney inflammation can be caused by direct (e.g., autoantibodies targeting renal antigens) or indirect (e.g., immune complex formation, complement activation, neutrophil extracellular trap formation) pathways. In both mechanisms, kidney inflammation subsequently leads to a loss of immune homeostasis, which facilitates further damage and the development of CKD. An uncoordinated tissue repair leads to fibrosis which compromises immune homeostasis. NETs = neutrophil extracellular traps; MAC = membrane attack complex (initiated by complement pathway activation).

Significant knowledge gaps exist regarding immune-mediated kidney disease, particularly glomerular diseases, which impact the management of these patients and can lead to detrimental outcomes. Serial kidney biopsies are often warranted to assess diagnosis, disease state (active inflammation vs. chronic injury), treatment response, and prognosis. Unfortunately, current histopathological techniques applied in routine practice sometimes fail to adequately differentiate between the distinct subtypes of glomerular diseases. There is a lack of widely-available, performant, and validated liquid (i.e., non-invasive) biomarkers that can inform treating physicians on disease-specific changes within the kidney. Most immune-mediated glomerular diseases are currently managed with broad-spectrum immunosuppressive therapies, which often cause side effects impacting patient quality of life, without adequate differentiation based on the underlying etiology. This approach underscores the critical need for the development of more targeted therapeutic options and the identification of biomarkers that can guide treatment decisions. Such advancements would enable personalized management strategies that could improve patient outcomes while minimizing adverse effects. Thus, the main objective of the current Research Topic was to report on and optimize long-term outcomes for children with immune-mediated kidney disease.

The burden of CKD in adolescents and young adults is underestimated. Sun et al. reported a significant increase in the global incidence of early-onset CKD in the past three decades. Nonetheless, the disability-adjusted life years rate remained stable, whereas mortality rates have decreased. Childhood-onset systemic-onset lupus erythematosus (cSLE) patients often exhibit more aggressive disease compared to adult patients, characterized by a higher incidence and more severe course of lupus nephritis (5, 6). The poor outcomes of cSLE patients are demonstrated in the study by Chen et al. Whereas up to 60.2% of the cSLE patients reached clinical remission during follow-up, only 3.5% reached complete remission (clinical and serological remission and immunosuppressant-free) and 19% reached steroid-free remission. Long-term remission was reached by only a minority of the patients. Crescentic GN encompasses a histopathological phenotype which can be observed in multiple GN subtypes, such as anti-neutrophil cytoplasmic antibodies (ANCA)-associated vasculitis (AAV). Zhang et al. compared outcomes of crescentic GN patients in the context of their etiology. Renal survival rates were lowest in patients with anti-GBM disease. These patients demonstrated more severe clinical manifestations and higher crescent scores on histopathology. AAV patients also had lower survival rates compared to the other GN subtypes presenting with crescents on histopathology. Borovitz et al. reported on C3 glomerulopathy relapse after kidney transplantation. In their case series of 19 C3 glomerulopathy patients, five underwent a kidney transplantation. Strikingly, all five patients experienced a relapse post-transplantation, which implies higher recurrence rates than previously believed. However, these results warrant validation in larger cohorts.

Three studies reporting on non-invasive biomarkers in glomerular diseases were incorporated in this Research Topic. In a prospective study by Zhaoyang et al. high pre-treatment serum levels of leptin and CCL22 were associated with steroid resistance in idiopathic nephrotic syndrome in childhood. Jiang et al. reported on vitamin D insufficiency in cSLE patients and its link with disease activity through changes in T helper 17 cells and regulatory T cells. Finally, Cody et al. tested the storage stability of six urinary biomarkers included in the Renal Activity Index for Lupus composite score.

The current Research Topic also encompasses two interesting case reports highlighting the difficulties in adequately discriminating between different GN subtypes. Kuang et al. reported the case of an eight-year-old girl with a myeloperoxidase-ANCA positive AAV initially presenting as a post-streptococcal acute GN. Daneshgar et al. described a case of C3 glomerulopathy relapse post-renal transplantation, complicated by a COVID-19 induced immune-complex mediated GN with membranoproliferative features and cryoglobulinemia.

Collectively, the papers incorporated in this Research Topic underscore the existing unmet needs in the diagnosis and management of children with immune-mediated kidney disease. Notwithstanding long term kidney outcomes have improved over the past decades, the prognosis for these children remains poor. These studies may act as a starting point to further optimize outcomes for children with immune-mediated kidney disease, particularly those with glomerular diseases. Future research should prioritize the discovery of novel liquid and clinical biomarker profiles that can inform physicians on diagnosis, disease state, prognosis, and treatment response, as well as possible new therapeutic targets.

## References

[1] Chadban SJ Atkins RC. Glomerulonephritis. Lancet. (2005) 365(9473):1797–806. 10.1016/S0140-6736(05)66583-X 15910953

[2] Keskinyan VS Lattanza B Reid-Adam J. Glomerulonephritis. Pediatr Rev. (2023) 44(9):498–512. 10.1542/pir.2021-005259 37653138

[3] GBD Chronic Kidney Disease Collaboration. Global, regional, and national burden of chronic kidney disease, 1990–2017: a systematic analysis for the global burden of disease study 2017. Lancet. (2020) 395(10225):709–33. 10.1016/S0140-6736(20)30045-3 32061315 PMC7049905

[4] Tecklenborg J Clayton D Siebert S Coley SM. The role of the immune system in kidney disease. Clin Exp Immunol. (2018) 192:142–50. 10.1111/cei.13119 29453850 PMC5904695

[5] Brunner HI Gladman DD Ibanez D Urowitz MD Silverman ED. Differences in disease features between childhood-onset and adult-onset systemic lupus erythematosus. Arthritis Rheum. (2008) 58(2):556–62. 10.1002/art.23204 18240232

[6] Hersh AO von Scheven E Yazdany J Panopalis P Trupin L Julian L Differences in long-term disease activity and treatment of adult patients with childhood- and adult-onset systemic lupus erythematosus. Arthritis Rheum. (2009) 61(1):13–20. 10.1002/art.24091 19116979 PMC2875186

